# Characterization and Quantification of Naphthenic Acids in Produced Water by Orbitrap MS and a Multivariate Approach

**DOI:** 10.1021/jasms.4c00172

**Published:** 2024-08-24

**Authors:** Jussara Valente Roque, Marcella Ferreira Rodrigues, Gabriel Henry M. Dufrayer, Iris Medeiros Júnior, Rogério Mesquita de Carvalho, Gesiane da Silva Lima, Gabriel Franco dos Santos, Boniek Gontijo Vaz

**Affiliations:** † Institute of Chemistry, Federal University of Goiás, Goiânia, GO 74690-900, Brazil; ‡ CENPES, PETROBRAS, Rio de Janeiro, RJ 21941-915, Brazil

## Abstract

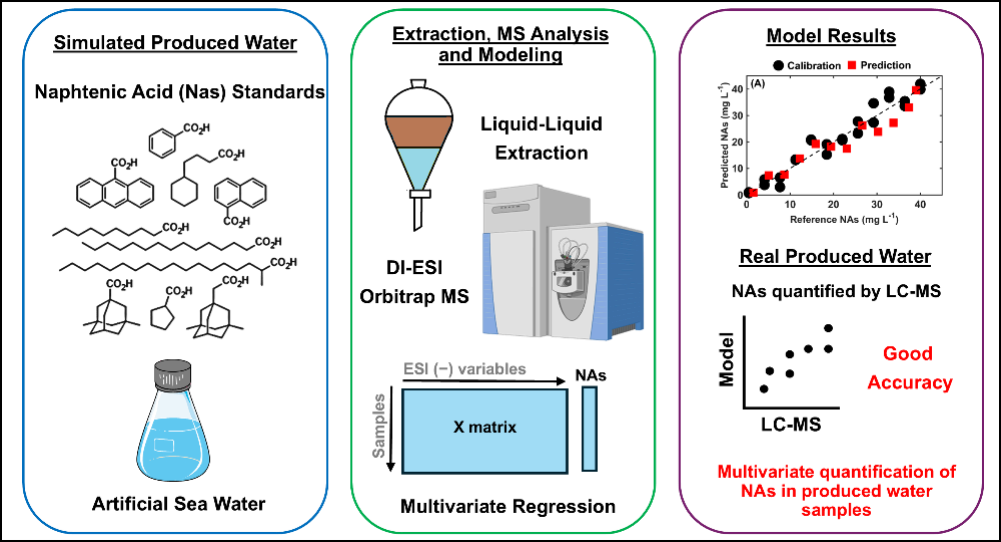

Naphthenic acids (NAs) naturally occur in crude oil and its associated produced water, presenting significant challenges, such as corrosion, in refinery apparatus and ecotoxicity in aquatic habitats. This study delineates a multivariate method to quantify NAs in produced water via electrospray ionization coupled with high-resolution Orbitrap mass spectrometry (ESI-Orbitrap MS). By employing liquid–liquid extraction, followed by direct infusion ESI(−)-Orbitrap MS, we characterized and quantified NAs employing a partial least-squares regression (PLS) model enhanced by the ordered predictor selection (OPS) algorithm. Thirty-six produced water samples were utilized, with 24 allocated for calibration and 12 designated for validation. The PLS-OPS model demonstrated notable accuracy in predicting NA concentrations in simulated and actual produced water samples ranging from ∼30 to 300 mg·L^–1^. This methodology offers a rapid yet robust alternative for quantifying NAs using mass spectrometry augmented by PLS and the OPS. Its significance is underscored by its potential to equip the petroleum industry with a swift and reliable monitoring mechanism for NAs in produced water, thereby aiding in mitigating environmental and operational risks.

## Introduction

Produced water is an abundant wastewater generated from offshore petroleum production and is a complex mixture containing different chemical substances, including heavy metals, hydrocarbons, phenols, additives, and organic acids, often represented as naphthenic acids (NAs);^1−4^ however, its composition can change according to the field’s geographical and geological factors.^5^

Naphthenic acids (NAs) are natural compounds present in crude oils and their effluents whose concentrations can vary intensely depending on the source of oil. Chemically, these compounds comprise a complex mixture of acyclic, cycloaliphatic, and aromatic carboxylic acids. The empirical formula for these compounds is C_*n*_H2_*n*+*Z*_O_2_, where *n* indicates the number of carbon and *Z* is zero or a negative number representing the hydrogen deficiency number.^3,6−12^ Recently, this definition has been expanded to include a complex mixture with more oxygen atoms.^6,13^

NAs are of concern in the petroleum industry due to several problems they cause, such as corrosion in refinery units, formation of emulsion, and production deposits, which have significantly impacted the productivity and increased costs of its systems.^14−17^ In addition, the NAs present in produced water are considered toxic mainly for aquatic systems, and their presence in the environment has been associated with various factors and different responses in a range of organisms, including plants, fish, rats, and bacteria.^3,5,9,17−20^ Considering the substantial environmental and economic impacts of NAs, using analytical methods and techniques for their characterization becomes crucial.

Since the development of ultrahigh resolution mass spectrometry (UHRMS) in the past few years, complex matrices such as petroleum and derivatives could be characterized by their thousands of compounds. Many studies in the literature have reported the potential of UHRMS in the characterization of NAs in crude oil,^8,13,14^ produced water,^4,6,13,21,22^ or other petrochemical wastewater.^23−26^ These characterization have been performed using different mass spectrometers, such as Orbitrap and FT-ICR, and several ionization techniques including Electrospray Ionization (ESI),^4,6,22,25^ Electron Impact Ionization (EI),^21^ Atmospheric Pressure Photoionization (APPI),^26,27^ Matrix-Assisted Laser Desorption/Ionization (MALDI),^14^ and Wooden-Tip Electrospray Ionization (WT-ESI).^13^

Even though UHRMS has shown a wide range of applications in characterizing NAs, quantifying NAs using UHRMS has revealed a more significant issue. In recent years, Samanipour and co-workers have studied the quantification of NAs from produced water using liquid chromatography (LC) coupled to ESI-MS, where a technical mixture of NAs was used as a standard and concentration of five produced water samples were found to range between 5 to 60 mg L^–1^ approximately.^3^ Hindle and co-workers also performed a similar study, where two technical mixtures were used as a standard for quantifying seven oil sands process-affected water (OSPW).^28^ Furthermore, previous studies from our group have reported the capability of quantifying NAs using different approaches, such as WT-ESI^13^ and eletromembrane extraction, followed by direct infusion ESI-MS.^4^ On the other hand, no study has reported a multivariate approach for quantifying NAs.

The quantification of NAs in produced water presents a significant analytical challenge, exacerbated by the complexity of the sample matrix and the diversity of NA compounds.^29−31^ Traditional methods for quantification, as highlighted in previous studies, have primarily relied on direct analytical techniques and standards-based approaches, which, while effective, may not fully account for the complexities inherent in environmental samples. This gap underscores the necessity for innovative approaches to accommodate the multifaceted nature of produced water and provide accurate, reliable quantification of NAs.^28,32^

The emergence of multivariate approaches heralds a transformative evolution in analyzing complex environmental samples. Multivariate statistical methods, such as partial least-squares regression (PLS) combined with ordered predictors selection (OPS), offer a sophisticated framework for tackling the intricacies of NA quantification.^33,34^ Unlike univariate methods, which consider single variables independently, multivariate techniques analyze multiple variables simultaneously, capturing the inherent relationships and patterns within the data.^35^ This holistic view is particularly advantageous in produced water, where the interaction between various compounds can significantly influence the analytical outcomes.

PLS regression, a cornerstone of multivariate analysis, is well-suited for quantifying NAs due to its ability to handle highly collinear and complex data sets.^36^ Focusing on the covariance between the dependent and independent variables allows the PLS to extract the most relevant information for predicting NAs concentrations, even in a noisy or highly complex background. This feature makes it an invaluable tool for environmental chemists seeking to quantify NAs precisely and accurately.

The integration of the OPS with the PLS further enhances the robustness of the quantification process. OPS is a variable selection method that systematically identifies the most predictive variables, optimizing the PLS model.^34^ In the context of NAs quantification, the use of OPS can help to distill the vast array of mass spectrometry data into a manageable subset of predictors, significantly improving model performance and interpretability. Therefore, this combination of PLS and the OPS represents a powerful multivariate approach that can overcome the challenges posed by the sample complexity and variability in produced water.

For the first time, this study proposes a multivariate approach for quantifying NAs in produced water, leveraging the strengths of PLS regression combined with OPS. This study proposes a new strategy for quantifying NAs compounds from produced water. Using LLE and direct infusion ESI(−)-Orbitrap MS, the proposed methodology led to a straightforward, simple, and fast approach to extract and analyze NAs in wastewater, enabling an efficient characterization and quantification when followed by a partial least-squares regression (PLS) combined with the ordered predictor selection method (OPS).

## Methods and Materials

### Chemicals and Materials

Ultrapure water was obtained with a water purification system (Master System MS2000, Gehaka, São Paulo, Brazil). HPLC grade methanol was provided by Tedia (Fairfield, U.S.A.). Ammonium hydroxide (NH_4_OH), hydrochloric acid, cyclopentane-carboxylic acid, benzoic acid, cyclohexanebutyric acid, 1-naphthoic acid, 9-anthracenecarboxylic acid, pentadecanoic acid, 2-methyloctadecanoic acid, decanoic acid, and 3,5-dimethyladamantane-1-carboxylic acid were purchased from Sigma-Aldrich (St. Louis, U.S.A.). Myristic-*d*_27_ acid was obtained from Cambridge Isotope Laboratories (Tewksbury, U.S.A.).

### Artificial Seawater Production

Artificial Seawater (ASW) was prepared according to the standard practice of preparing substitute ocean water (ASTM D 1141-98).^4,37^ The ASW preparation was performed using the following reagents: 24.53 g L^–1^ of NaCl (Neon, Sao Paulo, Brazil), 5.2 g L^–1^ of MgCl_2_.6H_2_O (Biograde, San Francisco, U.S.A.), 4.09 g L^–1^ of Na_2_SO_4_ (Synth, Diadema, Brazil), 1.16 g L^–1^ of CaCl_2_ (Synth, Diadema, Brazil), 0.695 g L^–1^ of KCl (Berzog, Sao Paulo, Brazil), 0.201 g L^–1^ of NaHCO_3_ (Biograde, San Francisco, U.S.A.), 0.101 g L^–1^ of KBr (Synth, Diadema, Brazil), 0.027 g L^–1^ of H_3_BO_3_ (Vetec, Rio de Janeiro, Brazil), 0.025 g L^–1^ of SrCl_2_·6H_2_O (Sigma-Aldrich, St. Lous, U.S.A.) and 0.003 g L^–1^ of NaF (Art Lab, Campinas, Brazil).

### Samples Preparation

**Figure 1:**
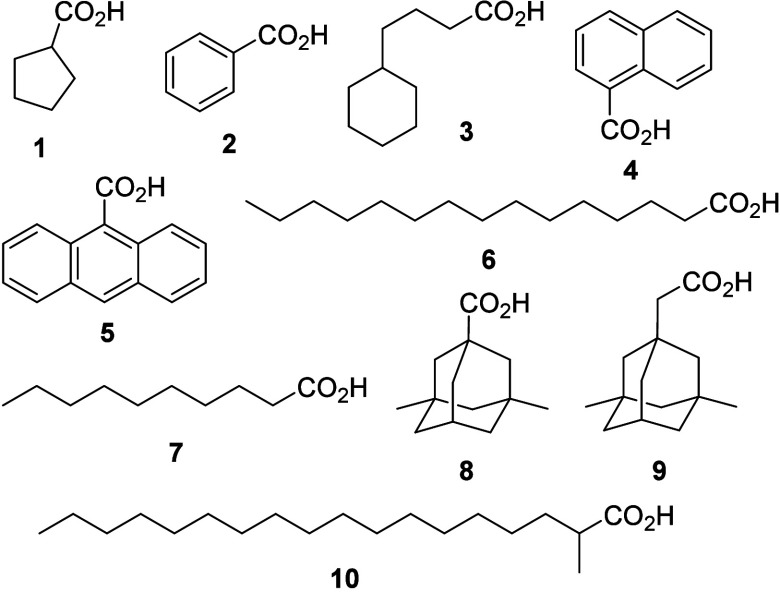
Chemical structures of ten carboxylic acid standards used in the simulated produced water with a concentration of 0.5 to 40 mg L^–1^.

In order to develop a multivariate regression model for NAs quantification, 36 simulated produced water samples were prepared using 10 carboxylic acid standards in a concentration range of 0.5 to 40 mg L^–1^. The NAs used were cyclopentanecarboxylic acid (**1**), benzoic acid (**2**), cyclohexanebutyric acid (**3**), 1-naphthoic acid (**4**), 9-anthracenecarboxylic acid (**5**), pentadecanoic acid (**6**), decanoic acid (**7**), 3,5-dimethyladamantane-1-carboxylic acid (**8**), 3,5-dimethyladamantane-1-acetic acid (**9**), and 2-methyloctadecanoic acid (**10**) (Figure 1). The stock solution of these compounds was prepared at a concentration of 10 g L^–1^ in methanol. The simulated produced water was individually prepared using 25 mL of ASM spiked with an equimolar mixture of all NAs standards at concentrations of 0.50, 4.10, 7.68, 11.27, 14.86, 18.45, 22.05, 25.63, 29.23, 32.82, 36.41, and 40.00 mg L^–1^. For each concentration, three simulated produced water samples were prepared, resulting in 36 sample solutions.

Twelve real produced water samples were also evaluated in this study. These samples were supplied by Petróleo Brasileiro S.A. (Petrobras).

### Liquid–Liquid Extraction

Liquid–liquid extraction (LLE) extractions were conducted using 25.0 mL of simulated produced water and real produced water samples, previously adjusted the pH for 2.0, and 25.0 mL of dichloromethane (3×) in a 125.0 mL separating funnel. Anhydrous sodium sulfate (6.0 g) was used to remove residual water, and the extract solvent was reduced using an IKA Rotary Evaporators RV 10 auto. The residual extract solution was concentrated using a SpeedVac Savant concentrator (Thermo Scientific, Asheville, U.S.A.). All extracts were kept stored at 4 °C until mass spectrometry analysis.

### ESI(−)-Orbitrap MS Analysis

The mass spectrometry analyses were performed using a HESI (heated electrospray ionization) ionization probe coupled to a Q-Exactive Orbitrap (Thermo Scientific, Bremen, Germany) in negative mode at 5.0 μL min^–1^ using a Chemyx Fusion syringe pump (Stafford, U.S.A.). The full scan was established in the mass range *m*/*z* 100 to 600 with parameters of spray voltage of 3.2 kV, capillary temperature of 275 °C, S-lens 100, and sheath gas of 5. For real produced water samples characterization, all spectra were processed using Composer software (Sierra Analytics, Modesto, U.S.A.) for molecular formula assignment.

### Multivariate Quantification

ESI(−)-Orbitrap MS data obtained in .*raw* files were converted to .*cdf* and imported into Matlab R2020a (MathWorks, Natick, MA, U.S.A.), where multivariate models were developed. A data matrix containing the intensity values, which are the independent variables, was constructed and is called matrix **X**. The rows of matrix **X** correspond to the 36 mixtures of NAs, and the columns correspond to *m*/*z*.

A vector containing the respective NAs concentrations of the simulated produced water solution was constructed and named **y**, the dependent variable. The vector **y** has a number of rows equal to the number of samples in the **X** matrix. To build the calibration models, PLS was used combined with variable selection by OPS. All calculations were performed in Matlab R2020a using the NewOPS package and personal algorithms.^34^

The use of PLS regression offers several significant advantages, particularly when dealing with complex matrices that contain various interfering substances. One of the foremost benefits of PLS regression is its ability to handle multicollinearity among predictor variables, which is common in spectral data, where many *m*/*z* values are highly correlated. PLS regression can efficiently extract relevant information from this correlated data by projecting it onto a new set of orthogonal components that maximize the covariance between the predictors and the response variable.^39^

Moreover, PLS regression excels in modeling data, even in the presence of interferents. By leveraging the entire spectral profile, PLS can differentiate between the signals of the target analytes and those arising from interfering substances. This capability is particularly valuable in our scenario, where samples often contain a complex mixture of compounds. The robust nature of PLS allows it to account for and correct these matrix effects, ensuring that the quantification of the target analytes remains accurate and reliable.^39,40^

Another key advantage of PLS regression is its capacity to handle large data sets with many variables and observations, making it suitable for high-dimensional data typical in mass spectrometry. PLS models can incorporate all relevant spectral information, providing a comprehensive analysis that captures the variability in the data due to both the analytes and the background matrix.^40^

Mean centering or autoscaling was used in all calculations to preprocess the **X** and **y** variables. Several types of normalizations were applied to the lines of the **X** matrix to find the best prediction model. The cross-validation method with random removal of three samples was used to perform this optimization and selection of the model’s latent variables (LV). Furthermore, in selecting variables, the OPS method was applied by using a window of 20 variables with an increment of 5, with 100% of the variables being tested.

The 36 sample set was divided into a calibration set (24 samples) and a prediction set (12 samples), separated randomly, with respect to the range modeled in both sets. The quality of the models was evaluated by the square root of the mean squared error (RMSE) and the correlation coefficient (*R*), which can be calculated using eqs 1 and 2, respectively.
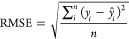
1
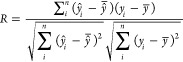
2In the equations, *y_i_* and  are measured and predicted values, respectively, of a sample *i*. Variables  and  are the averages of the measured and predicted values for a set of *n* samples. The RMSE and *R* are called RMSEC and Rc for calibration, RMSECV and Rcv for cross-validation, and RMSEP and Rp for prediction, respectively.

### LC-Orbitrap MS Analysis

The mass spectrometry analyses were performed using an HPLC-UV 1220 Infinity II (Agilent Technologies) coupled with a Q-Exactive Orbitrap instrument and a HESI source. The column used in this study was a Poroshell C18 column (4.6 mm × 100 mm × 2.7 μm). All samples were analyzed by using a gradient elution program. The binary mobile phases comprised A (water with 0.1% acetic acid) and B (methanol). The gradient elution started at 5% (B), kept constant for 1 min, linearly increased to 90% (B) in 8 min, followed by increasing to 100% (B) in 5 min, and kept constant for 16 min at 100% (B). The eluent was restored to the initial conditions in 6 min (36 min run). The flow rate was set at 0.5 mL min^–1^. The injection volume was 5 μL, and the column temperature was 40 °C. The ESI source conditions were established in the mass range *m*/*z* 100 to 600 with parameters of spray voltage 3.5 kV, capillary temperature 320 °C, S-lens 40, and sheath gas 5.

For the LC-MS quantification, the same ten carboxylic acid standards were used in the calibration curves at concentrations between 2.5 and 40 mg·L^–1^.

## Results and Discussion

### Characterization by ESI-Orbitrap MS

Before the method development for multivariate quantification of NAs from produced water, 12 produced water used in this study was extracted by liquid–liquid extraction and characterized by ESI(−)-Orbitrap MS. Figure S1 shows the 12 ESI(−)-Orbitrap MS spectra obtained in the *m*/*z* 100 to 600 range. For all spectra, the NAs compound peaks are most abundant between *m*/*z* 100 to 300, with high intensity in values below *m*/*z* 200.

**Figure 2:**
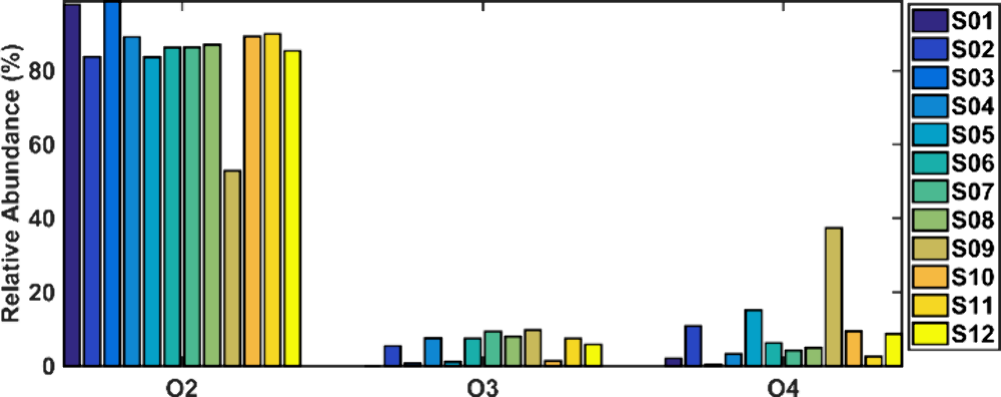
Class distribution of species obtained from the ESI(−)-Orbitrap MS analysis of the real produced water samples.

A class diagram was built to better understand the NAs in these samples. Figure 2 shows the samples’s class distribution obtained from the ESI(−)-Orbitrap MS analysis. Only one sample presents an abundance of an O2-containing compound below 80%, with an abundance of an O4-containing compound close to 40%. These results indicated that all 12 samples showed similar species distribution along with a high abundance of O2 species. Also, these class distributions are similar to NAs reported before.^4,13,22,38^

Figure S2 shows the DBE versus carbon number contour plots for NAs in the produced water samples. A similar distribution of compounds with DBE values between 1 to 10 and carbon numbers 5 to 20 were found. Only compounds with DBE 1 showed an extensive range of carbon numbers, with values up to 26. After the characterization of all NA extracts, a method for multivariate quantification was built.

### Multivariate Quantification

As a first step to the method development for NA multivariate quantification, 36 simulated produced water samples were prepared using ten carboxylic acid standards in a concentration range of 0.5 to 40 mg L^–1^, as described in the Methods and Materials. Each simulated produced water was extracted using the same extraction methodology applied to produced water and analyzed by ESI(−)-Orbitrap MS.

**Figure 3:**
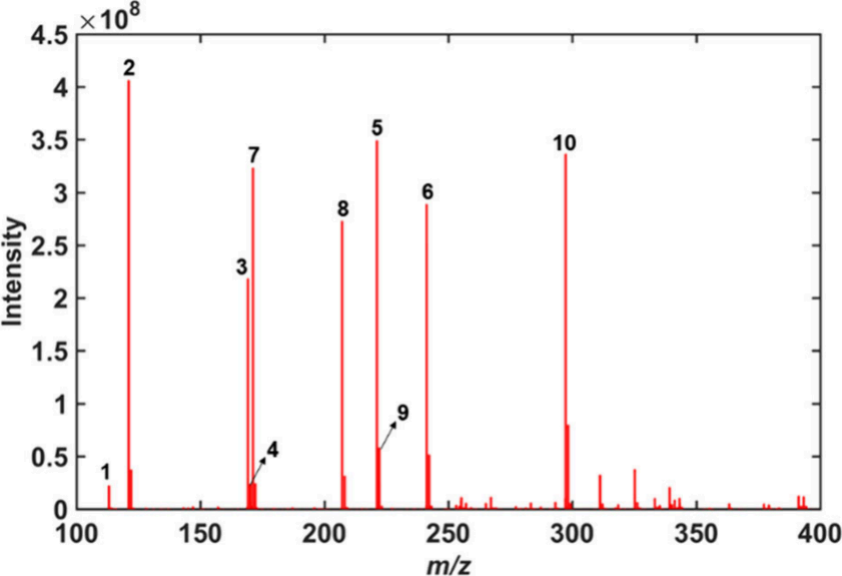
Average mass spectrum of the 36 mixtures of NAs obtained by ESI(−)-Orbitrap MS. Cyclopentanecarboxylic acid (**1** – *m*/*z* 113.06), benzoic acid (**2** – *m*/*z* 121.03), cyclohexanebutyric acid (**3** – *m*/*z* 169.12), 1-naphthoic acid (**4** – *m*/*z* 171.05), 9-anthracenecarboxylic acid (**5** – *m*/*z* 221.06), pentadecanoic acid (**6** – *m*/*z* 241.22), decanoic acid (**7** – *m*/*z* 171.14 3,5-dimethyladamantane-1-carboxylic acid (**8** – *m*/*z* 207.14), 3,5-dimethyladamantane-1-acetic acid (**9** – *m*/*z* 221.15), and 2-methyloctadecanoic acid (**10** – *m*/*z* 297.28).

From the alignment of the 36 spectra of the NAs mixtures, the spectra were organized in the **X** matrix to construct the multivariate model. This matrix was aligned considering a resolution of 0.001, thus obtaining 77187 variables from *m*/*z* 100 to 400. The average mass spectrum obtained from the 36 mixtures of NAs is shown in Figure 3.

Notably, despite our initial intention to analyze the full mass range of *m*/*z* 100–600, meticulous examination revealed an absence of discernible signals in both the standard mixtures and real produced water samples beyond the *m*/*z* value of 400. This intriguing observation prompted a deliberate decision to constrain the mass range, restricting subsequent analyses to *m*/*z* = 100–400.

Table 1 showcases the calculated parameters for the PLS-OPS model for quantifying NAs. The model was derived from the autoscaled **X** matrix, with intensities normalized by the total sum.

**Table 1:** Calculated Parameters for the PLS-OPS Model for NAs (mg L^–1^) Prediction^a^

pretreatment	auto scaling normalization by sum
LV	10
RMSEC	3.0683
Rc	0.9735
RMSECV	3.7541
Rcv	0.9539
RMSEP	3.6182
Rp	0.9652

a LV: number of latent variables. RMSE: root-mean-square error of calibration (RMSEC), cross-validation (RMSECV), and prediction (RMSEP). R: correlation coefficient of calibration (Rc), cross-validation (Rcv), and predction (Rp).

The analysis of the results reveals that the RMSE values are notably low concerning the modeled range (0.5–40 mg L^–1^), underscoring the model’s robustness and potential applicability in predicting NAs. The PLS-OPS model demonstrates a praiseworthy predictive performance, as evidenced by the calibration and prediction correlation coefficients (Rc and Rp, respectively), which exceed 0.95. Such high correlation coefficients indicate a strong linear relationship between the predicted and reference values, confirming the model’s efficacy in accurately predicting NAs. Moreover, the relatively low RMSEs further attest to the model’s accuracy and capability to minimize prediction errors within the specified concentration range.

**Figure 4:**
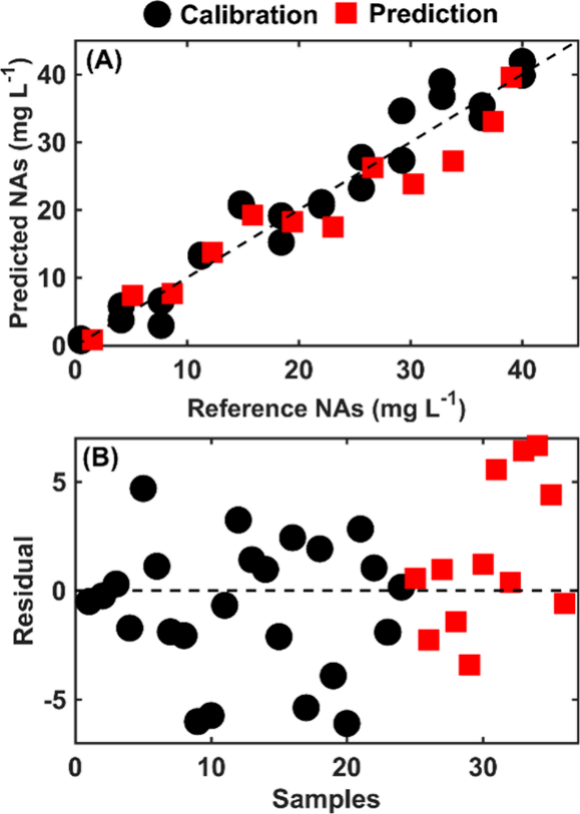
(A) Scatter plot of the reference NAs versus predicted NAs for calibration (●) and prediction (■) sets, demonstrating the model’s accuracy in estimating NAs concentrations. (B) Residuals plot for both calibration (●) and prediction (■) sets.

Figure 4 displays the reference values, predicted values, and residuals for the 36 sample mixtures. Ideally, all points should align with the diagonal line depicted in Figure 3A; the closer a point is to this line, the more accurate is the prediction relative to the reference value. This visual representation underscores the accuracy of the predictions and highlights the relationship between the predicted and actual concentrations of NAs.

An overfitting model may not be advantageous, as it could be excessively tailored to the specific data set it was trained on, potentially resulting in more significant prediction errors when applied to new data sets. Therefore, striking a balance between the model fit and prediction errors for samples not included in the training phase is paramount. As observed, the random distribution of residuals suggests the absence of systematic errors in the model. This lack of pattern in the residuals indicates that the model can handle bias and uncorrected trends, which could compromise its predictive accuracy.

The implications of these findings are 2-fold. First, the PLS-OPS model exhibits a commendable level of accuracy in predicting NAs, as evidenced by the proximity of predicted values to the reference line in Figure 4A. Second, the random distribution of residuals corroborates the model’s reliability and generalizability to new data. It highlights the model’s ability to maintain its predictive performance without being overly specialized to the training data set, making it a versatile tool for predicting NAs concentrations across various contexts. The absence of systematic errors further enhances the model’s utility, providing confidence in its predictions and underscoring its potential for widespread application in analytical chemistry.

**Figure 5:**
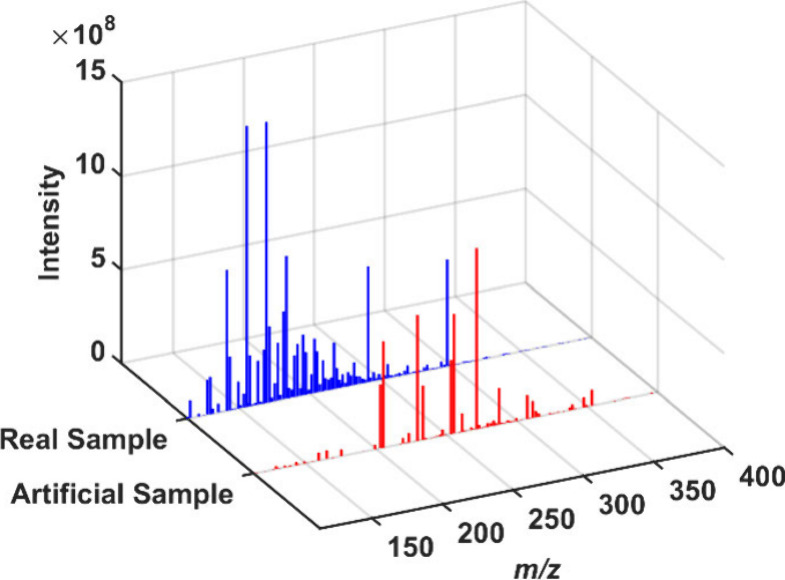
Mass spectra comparison between artificial and real produced water samples obtained by ESI(−)-Orbitrap MS.

The model’s accuracy was further evaluated through its application to 12 real-produced water samples of produced water. In Figure 5, the spectral analysis juxtaposes a spectrum from the modeled mixture set with the spectrum of actual, real-produced water samples. A noteworthy observation from the spectral comparison is the substantially higher number of signals in the produced water sample than in the modeled artificial sample. This disparity underscores the complexity of real-world samples and the plethora of interfering substances that may be present.

An immense variety of peaks in the produced water spectrum indicates a diverse range of compounds, potentially affecting the prediction of NAs. Despite the increased complexity, the strength of the PLS-OPS model lies in its ability to discern the relevant patterns associated with NAs amidst a background of numerous other signals. Table 2 details the predicted values for NAs in real-produced water samples using the PLS-OPS model, offering insight into the model’s capacity to cope with complex, real-world analytical challenges.

**Table 2:** Comparison of Predicted NAs by PLS-OPS Model with Reference Values^a^

sample	PRED1	PRED2	LC-MS	difference
**S01**	4.89	97.80	84.48	13.32
**S02**	12.09	241.80	209.52	32.28
**S03**	3.12	62.40	61.50	0.90
**S04**	16.02	320.40	308.61	11.79
**S05**	8.19	163.80	158.73	5.07
**S06**	1.56	31.20	28.28	2.92
**S07**	2.03	40.60	35.75	4.85
**S08**	1.98	39.60	35.28	4.32
**S09**	1.87	37.40	35.8	1.60
**S10**	9.47	189.40	165.6	23.8
**S11**	2.09	41.80	35.64	6.16
**S12**	3.49	69.80	62.28	7.52

a PRED1 = Predicted values in mg L^–1^ by PLS-OPS; PRED2 = Predicted values corrected by dilution factor (20×); LC-MS = liquid chromatography–mass spectrometry used as the reference method.

In the pursuit of accurate quantification of NAs in real produced water samples, we employed a developed LC-MS methodology emanating from our laboratory’s innovative analytical techniques. LC-MS, renowned for its unparalleled accuracy and precision, is the benchmark for evaluating our multivariate PLS-OPS model with ESI(−)-Orbitrap MS.

Upon dilution of produced water samples by a factor of 20, essential for accommodating the ESI(−)-Orbitrap MS analysis, the imperative adjustment of this dilution was meticulously accounted for in comparison with LC-MS data. This careful recalibration laid the groundwork for a robust comparative analysis, encapsulating the outcomes in Table 2. The data vividly illustrate the congruence between the corrected PLS-OPS model predictions and established LC-MS measurements. Notably, the strong correlation coefficient of 0.99, albeit with an average difference of 9.5 units, points to the fidelity of the multivariate model in mirroring the reference method’s outcomes.

While the near unity in correlation speaks volumes about the multivariate method’s potential, the observed discrepancy in predicted values prompts a discussion on potential model biases. A plausible source of such bias could be the presence of interferents in real-produced water samples, which may influence the model’s predictive accuracy despite their absence in artificial mixtures. The profusion of additional signals in real-produced water samples could be the model’s problem, contributing to the slight variance observed.

Several factors could account for the systematic difference between the predicted NA concentrations (dilution corrected) and the values obtained by LC-MS. First, our direct infusion HRMS method may have different matrix effects and ionization efficiencies compared to LC-MS. While HRMS relies on the simultaneous direct ionization of all components in the sample, LC-MS involves separation before detection, which can affect the calibration and quantitation of the NAs. Additionally, differences in sample preparation, extraction efficiency, recovery, and dilution process between the two methods can contribute to the observed differences.

However, these findings should be consistent with the substantial promise that multivariate models exhibit. The alignment of the model’s predictions with the high-resolution LC-MS measurements, even when challenged with complex sample matrices, signals a breakthrough in the applicability of such models for quantitative analysis in environmental monitoring. We must delve deeper into these multivariate methods, refining and validating them, to harness their full potential for quantifying NAs in produced waters.

## Conclusion

The present study showed a new approach to quantify NAs by direct flow ESI(−)-Orbitrap MS. As a first step, a method was developed and validated for quantifying NAs standards in simulated produced water. Using direct flow ESI(−)-Orbitrap MS of 36 simulated produced water samples, the PLS-OPS model exhibited a notable accuracy level in predicting NAs, observed by the proximity of predicted values to the reference line. The model’s robustness was further shown through the method application to 12 real-produced water samples obtained from offshore petroleum exploitation on the Brazilian coast, where the predictable results were very similar to the obtained values by LC-MS. Notably, the strong correlation coefficient of 0.99, though with an average difference of 9.5 units, points to the fidelity of the multivariate model in mirroring the reference method’s outcomes. Additionally, using a fast and straightforward methodology, this approach could be easily applied to quantify NA compounds in several wastewater samples. Therefore, this research offers a significant advancement in analytical methods for quantifying NAs, tailored explicitly to monitoring produced water in the petroleum industry, where accurate assessment of water quality is critical for environmental management and compliance with regulatory standards.

## Supporting Information Available

ESI(−)-Orbitrap MS spectra of the produced water sample extracts (Figure S1). DBE and carbon number distribution of O2-containing compounds by ESI (−)-Orbitrap MS analysis for real produced water samples (Figure S2) (PDF)
