# Supplementary material for: Characterization and Quantification of Naphthenic Acids in Produced Water by Orbitrap MS and a Multivariate Approach
Source: J Am Soc Mass Spectrom. 2024 Aug 24;35(9):2128–35. doi: 10.1021/jasms.4c00172 (PMC11378276; doi:10.1021/jasms.4c00172)
Supplement: Supplementary file 1 — js4c00172_si_001.pdf [file js4c00172_si_001.pdf]

Supporting Information

Characterization and quantification of naphthenic acids in produced water by Orbitrap MS and multivariate approach

Jussara Valente Roque,[†] Marcella Ferreira Rodrigues,[†] Gabriel Henry M. Dufrayer,[†] Iris Medeiros Júnior,[††] Rogério Mesquita de Carvalho,[††] Gesiane da Silva Lima,[†] Gabriel Franco dos Santos,[†,*] Boniek Gontijo Vaz[†,*]

[†] Institute of Chemistry, Federal University of Goiás, Goiânia, 74690-900, GO, Brazil.

[††] CENPES, PETROBRAS, Rio de Janeiro, RJ, 21941-915, Brazil.

*Corresponding author.
E-mail address: boniek@ufg.br (B. G. Vaz)
E-mail address: gfs.dossantos@gmail.com (G. F. dos Santos)

Table of contents:

Figure S1. ESI(–)-Orbitrap MS spectra of the produced water sample extracts......**S2**

Figure S2. DBE and carbon number distribution of O2-containing compounds by ESI (–)-Orbitrap MS analysis for real produced water samples **S2**

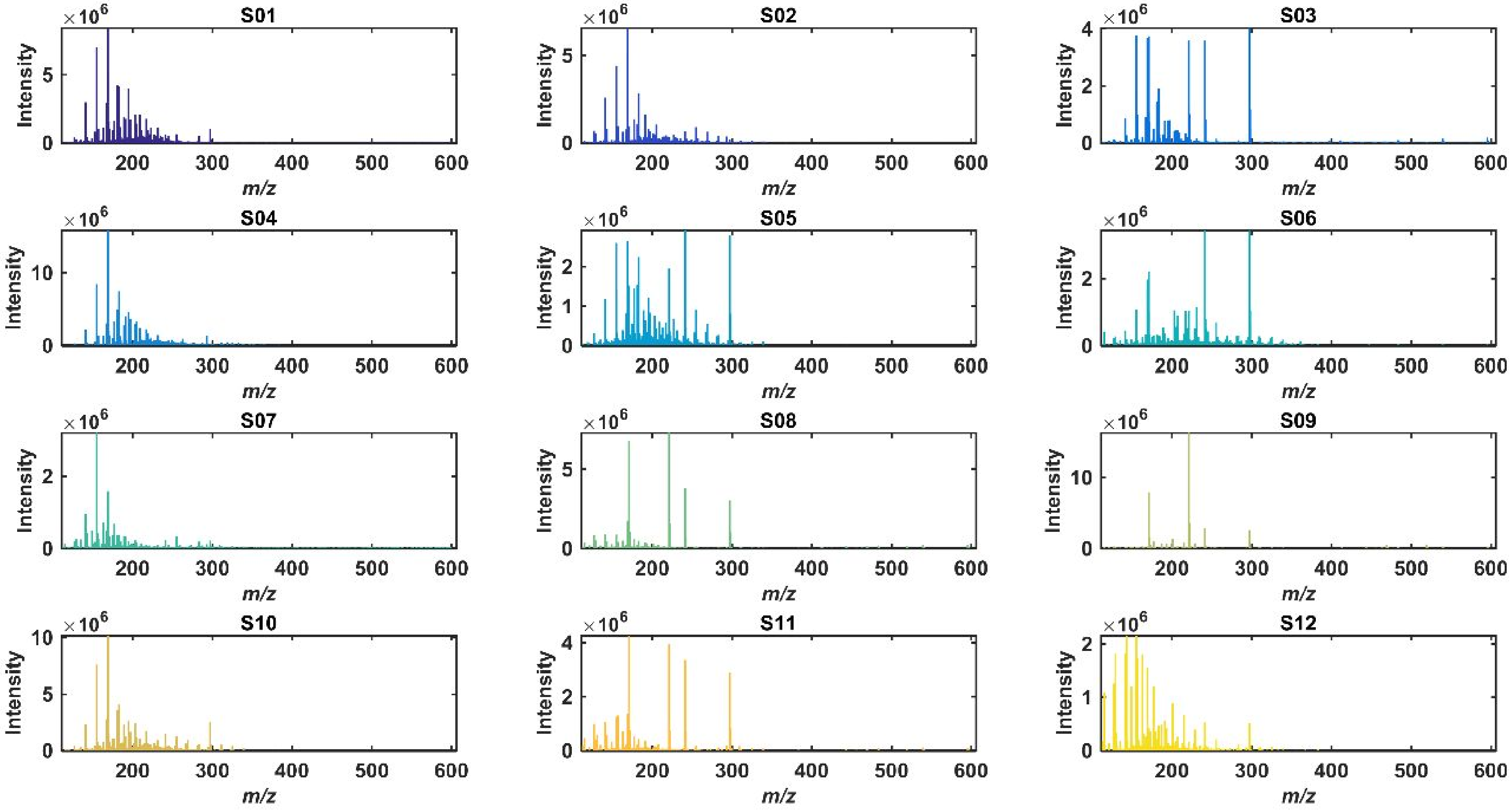

Figure S1. ESI(–)-Orbitrap MS spectra of the produced water sample extracts.

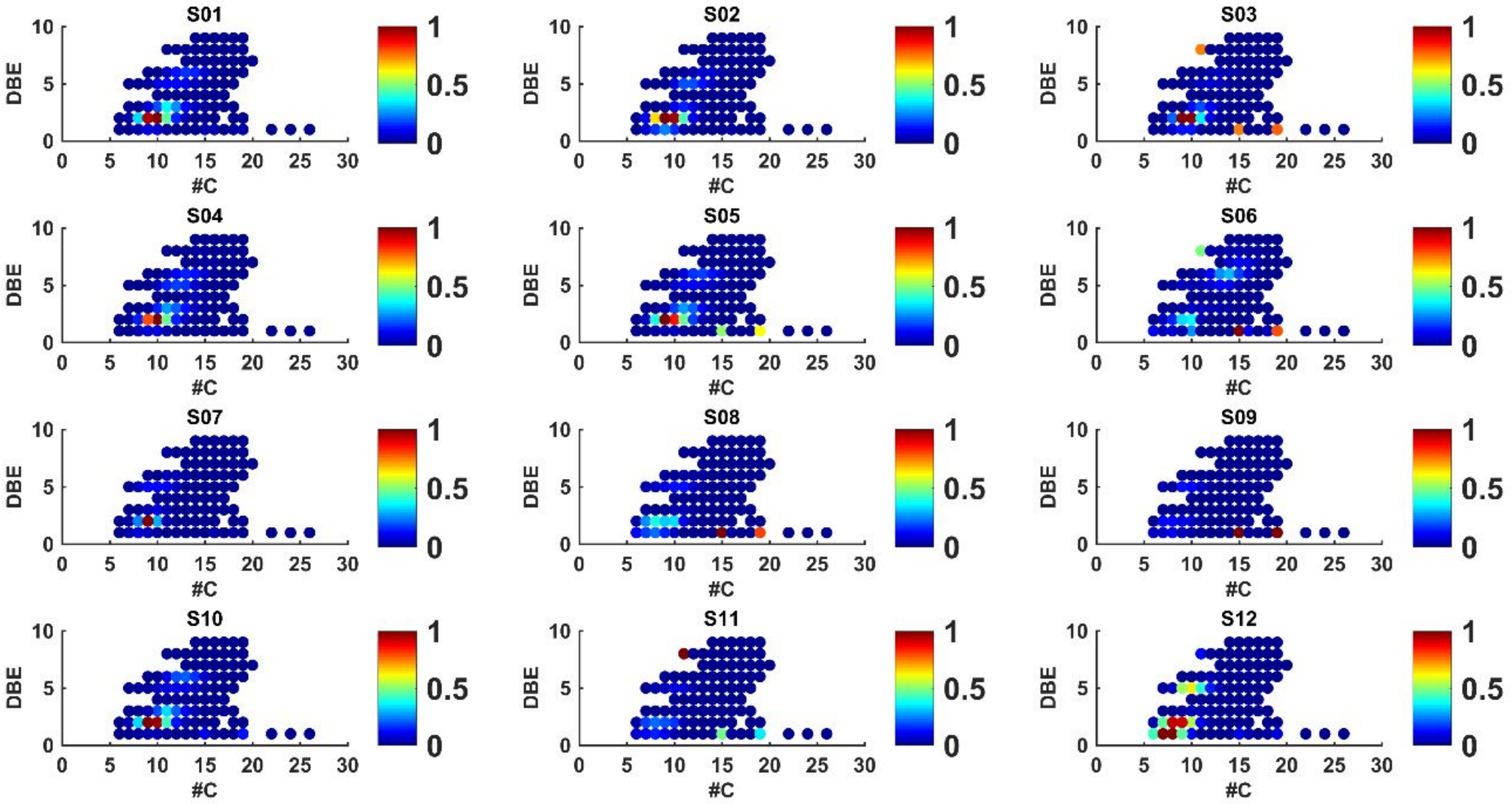

Figure S2. DBE and carbon number distribution of O2-containing compounds by ESI (–)-Orbitrap MS analysis for real produced water samples.
